# Effect of the *Ing Oun* pillow for supporting baby’s back in a side-lying position to promote breastfeeding: A proof-of-concept randomized controlled trial in Thailand

**DOI:** 10.18332/ejm/223935

**Published:** 2026-07-03

**Authors:** Pornsri Disorntatiwat, Sudjit Liblub, Streerut Thadakant

**Affiliations:** 1 Ramathibodi School of Nursing, Faculty of Medicine Ramathibodi Hospital, Mahidol University, Bangkok, Thailand

**Keywords:** breastfeeding, postpartum, mothers, interventions, normal birth

## Abstract

**INTRODUCTION:**

Despite the World Health Organization’s recommendation for exclusive breastfeeding, Thailand’s rate was only 14% in 2019. Many new mothers experience discomfort and lack confidence during breastfeeding. The novel *Ing Oun* breastfeeding pillow was developed to support breastfeeding in the side-lying position. This study compared breastfeeding effectiveness between the *Ing Oun* pillow and the normal side-lying position and evaluated maternal satisfaction.

**METHODS:**

A proof-of-concept randomized controlled trial was conducted in a postpartum unit in Thailand. Ninety-two first-time mothers with vaginal births were randomly assigned to either the *Ing Oun* breastfeeding pillow in the side-lying position (intervention) or the normal side-lying position (control). Breastfeeding effectiveness was assessed by nurse-midwives and mothers based on infant positioning and attachment. Maternal satisfaction with the pillow was measured using a self-report questionnaire.

**RESULTS:**

Compared with the control group, mothers using the *Ing Oun* breastfeeding pillow demonstrated significant improvements in breastfeeding effectiveness, including reduced nipple pain during suckling [median=3 (IQR: 2–4) vs 3 (IQR: 1–4), p<0.001], observing more areola above the infant’s top lip [median=4 (IQR: 3–4) vs 3 (IQR:1–4), p<0.001], and improved support for continued suckling [median=4 (IQR: 2–4) vs 3 (IQR: 1–4), p<0.05]. Mothers in the intervention group reported high levels of satisfaction with the pillow, with 65.2% expressing confidence in breastfeeding.

**CONCLUSIONS:**

The *Ing Oun* breastfeeding pillow shows potential as a breastfeeding support aid; however, further research is needed to refine its design and evaluate broader applicability.

**CLINICAL TRIAL REGISTRATION:** The study is registered on the official website of Thai Clinical Trials Registry.

**IDENTIFIER:** TCTR20241016002

## INTRODUCTION

Breast milk is the optimal source of nutrition for infants, offering complete nourishment and essential antibodies that protect against common childhood illnesses^1^. Breastfeeding fosters a strong bond between mothers and infants and enhances maternal self-esteem^1^. The World Health Organization recommends exclusive breastfeeding for the first six months and continuing breastfeeding for up to ≥2 years^2^. Despite these benefits, breastfeeding rates in Thailand remain low. According to the National Statistical Office of Thailand^3^, the exclusive breastfeeding rate at six months was only approximately 14%. This is significantly below the World Health Assembly’s target of achieving a 50% exclusive breastfeeding rate by 2025^4^.

According to the Ten Steps to Successful Breastfeeding, it is crucial for postpartum women to start breastfeeding within the first hour after birth. This early initiation helps stimulate the production of prolactin, a hormone that is essential for milk production. Following this initial breastfeeding, it is recommended that mothers breastfeed their infants every 2–3 hours or on demand, aiming for approximately eight sessions per 24-hour period. This frequent breastfeeding helps to establish and maintain an adequate milk supply^1,5^. However, many mothers face challenges such as discomfort, a lack of confidence, and exhaustion, which can impede their ability to breastfeed successfully^6-8^. Inadequate breastfeeding can lead to insufficient milk production and a shorter breastfeeding duration, particularly for first-time mothers^6^. Effective attachment and positioning are key to ensuring that the baby can efficiently extract milk and that the mother remains comfortable throughout the breastfeeding process^9^.

The side-lying breastfeeding position, where both mothers and babies lie on their sides facing each other, can be beneficial, especially for exhausted mothers and those with limited breastfeeding experience^10^. This position involves using pillows to support the mother’s back and cradle the baby’s head, ensuring that the baby’s mouth is level with the nipple for easier milk intake^11^. However, achieving the correct alignment can be challenging, and additional support, such as a pillow or rolled blanket behind the baby’s back, may be required to prevent the baby from rolling away^10^. Currently, there are various tools designed to assist mothers with breastfeeding, including functional nursing bras and nursing pillows, which can aid in sustaining breastfeeding^7,12^. However, this innovation is used to support breastfeeding in the sitting position rather than the lying position.

Therefore, finding new ways to help and support first-time mothers in early breastfeeding comfortably and correctly to continue breastfeeding is a justification. The *Ing Oun* breastfeeding pillow was designed and developed by researchers to potentially provide a better breastfeeding position that is more suitable for both mothers and babies. This pillow is designed to support the baby’s back and prevent the baby from rolling away from the mother while breastfeeding in the side-lying position. The name *Ing Oun* comes from a Thai phrase meaning ‘warm embrace’ or ‘comforting support’, reflecting the pillow’s purpose of providing comfort and support for breastfeeding.

Our aims were to compare the effectiveness of the *Ing Oun* breastfeeding pillow in which first-time mothers are breastfeeding in a side-lying position and a normal side-lying position. Additionally, we aimed to evaluate the ability of the breastfeeding pillow to support breastfeeding in a side-lying position among first-time mothers. The null hypothesis was that there was no difference in mean breastfeeding effectiveness between using the breastfeeding pillow and breastfeeding in a side-lying position (primary outcome), with mothers’ satisfaction as a secondary outcome.

## METHODS

### Study design

This study was a proof-of-concept randomized controlled trial (RCT) designed to assess the effectiveness of the *Ing Oun* breastfeeding pillow in supporting breastfeeding in the side-lying breastfeeding position compared with the normal side-lying position in first-time mothers. Given that the pillow is still in its early stages of development, researchers have adopted a proof-of-concept approach. This approach is crucial during the initial phases of product design because it allows for the early identification of whether a product is capable of fulfilling its intended purpose^13^. The primary outcome of the study was breastfeeding effectiveness, which was assessed by both nurse-midwives and mothers. Breastfeeding effectiveness in this study was defined by the baby’s position (head and body in line, close to the mother, facing the breast with the nose opposite the nipple, body fully supported) and good attachment (more areola visible above the top lip, mouth wide open, lower lip turned outwards, and chin touching the breast). The secondary outcome was the mothers’ satisfaction with the *Ing Oun* breastfeeding pillow, which was measured via a self-report questionnaire. This study was registered with the Thai Clinical Trials Registry (TCTR20241016002).

The *Ing Oun* breastfeeding pillow was specifically designed and developed for this study, with a pilot test conducted to refine the innovation. The pilot testing informed several modifications to improve the functionality of the device. These refinements included refining the round pillows with a central indentation to ensure a consistent size and filling them with soft material to provide gentle support for the baby’s head. Beads were added to the filling to increase the weight and stability of the main pillow. The outer fabric was also changed from cotton to water-resistant fabric to improve durability and ease of cleaning. Following the design phase, the research protocol and assessment procedures were demonstrated step-by-step to the research assistants for 30–60 minutes. These assistants then performed the assessment process to verify its validity. Given that the study involved an innovation in its early stages, the *Ing Oun* breastfeeding pillow was exclusively used in the postpartum unit throughout the study period.

### Intervention group

**Figure 1:**
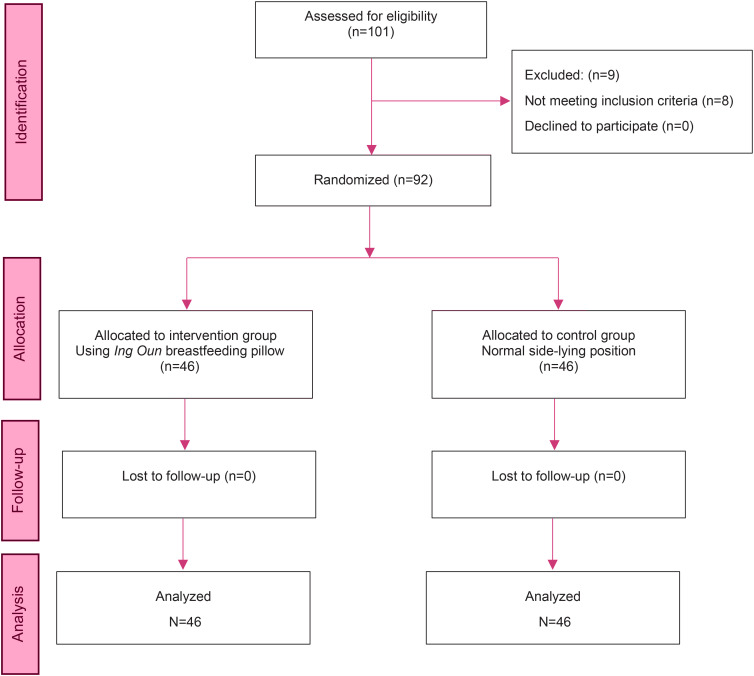
The experimental flowchart

The participants in the intervention group received support via the *Ing Oun* breastfeeding pillow during the side-lying position. A research assistant assisted the mother in placing the pillow behind the baby’s back in the side-lying position. Once the baby began sucking, the research assistant guided the mother in adjusting the pillow for a firm hold. The mother then continued to breastfeed for 15 minutes. The research assistants observed and measured the effectiveness of breastfeeding. Following the breastfeeding session, mothers were asked to complete questionnaires assessing both the effectiveness of breastfeeding and their satisfaction with the *Ing Oun* breastfeeding pillow (Figure 1).

### Control group

Participants in the control group received support for breastfeeding in a normal side-lying position. A research assistant provided assistance during a 15-minute breastfeeding session, and effectiveness was similarly observed and measured. After breastfeeding, mothers were asked to complete a questionnaire assessing the effectiveness of breastfeeding (Figure 1).

### Setting and participants

The study was conducted from January to July 2024 in the postnatal unit of Ramathibodi Hospital, Bangkok, Thailand. This hospital delivers nearly 800 babies annually, with approximately 280 vaginal births, of which 200 are from first-time mothers. Two hours post-delivery, mothers were transferred to the postnatal unit, where they received care and support. Healthy mothers and their newborns are typically discharged within 48–72 hours from the postnatal unit. Breastfeeding support during the postpartum period was provided by nurse-midwives, who also served as research assistants owing to their expertise in assessing and observing breastfeeding behavior.

A convenience sampling approach was used to recruit mothers who had vaginal births. The inclusion criteria for the study were first-time mothers with babies weighing at least 2500 g, no tongue-tie or post-delivery complications, normal nipple size (at least 0.5 cm), and proficiency in reading Thai. The research team approached mothers who met these criteria within the first 72 hours post-delivery while they were still in the hospital, inviting them to participate. The exclusion criteria included babies with serious health conditions requiring additional care, such as respiratory distress syndrome, hypothermia, or jaundice requiring phototherapy; mothers with postpartum complications; and those who refused or withdrew from participation.

To minimize variability in breastfeeding assistance, each mother received consistent support from the same nurse-midwife throughout the study. Both the intervention and control groups received usual breastfeeding care provided by nurse-midwives, including guidance to breastfeed every 2–3 hours or whenever the infant showed signs of hunger.

### Recruitment and data collection

Recruitment and data collection were conducted daily in the postpartum unit. The participants were randomly assigned to either the intervention group or the control group via simple randomization with a 1:1 allocation ratio. The allocation sequence was generated by a person not involved in the research via a computerized random number generator. The allocation was concealed from the researchers via sequentially numbered, sealed envelopes. Once a participant was enrolled, the researcher opened the next sealed envelope and assigned the participant to the indicated group.

### Sample size calculation

The sample size was calculated on the basis of a pilot study involving five mothers conducted in the same setting to test the study’s feasibility (SD=1.2). Using the formula for sample size calculation, considering an alpha error of 0.05, a delta of 0.5, and a test power of 80% under a two-tail hypothesis, the sample size was determined to be 92 mothers in each group^14^.

### Intervention

**Figure 2:**
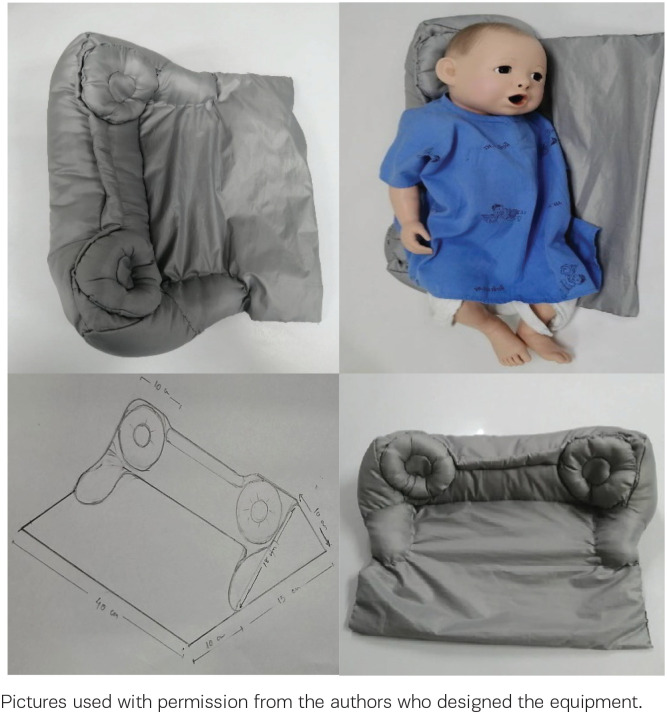
Design of the Ing Oun breastfeeding pillow (left) and during the use of the pillow to support the side-lying position adopted (right)

The *Ing Oun* breastfeeding pillow is made from water-resistant rubber fabric and consists of two parts (Figure 2). The first part is the main pillow, which has a triangular shape, measuring 15×18×10 cm, with a length of 40 cm. It is filled with fabric, synthetic fibers, and beads to provide weight, offering support for the baby’s back and preventing it from rolling during breastfeeding. Additionally, there are two flat, round pillows, each with a central indentation resembling a doughnut, measuring 10 cm in diameter on both sides. These smaller pillows are filled with synthetic fibers to provide support to the baby’s occipital area, ensuring that the baby’s head rests comfortably. The second part of the pillow is rectangular, measuring 10×40 cm, and is designed to support the baby’s body. The joint where the two pillow sections meet is approximately 7 cm thick, providing extra support for the baby’s neck (Figure 2).

During the intervention, the research assistant taught mothers how to breastfeed in the side-lying position and assisted in placing the *Ing Oun* breastfeeding pillow behind the baby’s back. This position replaced the mother’s arm, helping to bring the baby’s body close to the mother and aligning the baby’s mouth with the mother’s breast for a better latch. This positioning also minimized shoulder fatigue for the mother while they were breastfeeding.

### Measurement

Breastfeeding outcomes can be evaluated from different perspectives. In this study, breastfeeding effectiveness refers to observable indicators of breastfeeding performance, including appropriate infant positioning, effective attachment, and the infant’s ability to sustain suckling during feeding^15^. These indicators reflect the quality of the breastfeeding process and are consistent with commonly recognized clinical breastfeeding assessment criteria used in practice. Direct observation of breastfeeding sessions, including before and after infant attachment, enables healthcare providers to identify potential difficulties and provide support for effective breastfeeding^15^. Alongside breastfeeding effectiveness, maternal satisfaction is also an important outcome when evaluating new breastfeeding interventions. In this study, maternal satisfaction refers specifically to mothers’ experiences when using the breastfeeding pillow, including aspects such as comfort, ease of use, and its ability to support the intended breastfeeding position. Assessing these factors helps determine whether the device is practical and acceptable for use in breastfeeding practice.

The effectiveness of breastfeeding was measured via a questionnaire developed on the basis of key aspects of effective breastfeeding, including four key points for baby positioning (head and body aligned, close to mother, facing the breast with the nose at the nipple, whole body supported) and good attachment signs (more areola visible above the upper lip, wide mouth opening, everted lower lip, chin touching the breast)^15^. Both the participants and the nurse-midwives completed questionnaires, which included five items ranked on a 5-point Likert scale ranging from 1=strongly agree to 5=strongly disagree.

The satisfaction questionnaire for the *Ing Oun* breastfeeding pillow was based on the diffusion of the innovation model, which considers compatibility, complexity, trialability, and observability as key factors influencing adoption^16^. The questionnaire comprises 14 items ranked on a 5-point scale ranging from lowest to highest satisfaction.

To ensure the validity and reliability of the questionnaires, a pilot study was conducted. Content validity was assessed by a panel of three experts with experience in maternal-newborn and midwifery research, leading to revisions for improved clarity. The questionnaires were piloted with five mothers (separate from the main study sample), and internal consistency was evaluated via Cronbach’s alpha, resulting in a coefficient greater than 0.8, indicating high reliability.

### Ethics

Ethical approval was obtained from the Ethics Committee on Human Subjects of the Faculty of Medicine at Ramathibodi Hospital, Mahidol University (Approval number: IRB COA. MURA2023/205; Date: March 2023)^17^. Participants provided informed consent.

### Statistical analysis

All data analysis was performed via IBM SPSS Statistics 27.0.1.0 software^18^. The Shapiro–Wilk test was used to determine the normality of the data distribution for quantitative variables. Descriptive statistics with means and standard deviations (SDs) were used to present the data. The Mann–Whitney U test was used to compare the effectiveness of breastfeeding scores between the two groups, with a p<0.05 considered statistically significant.

## RESULTS

**Figure 3:**
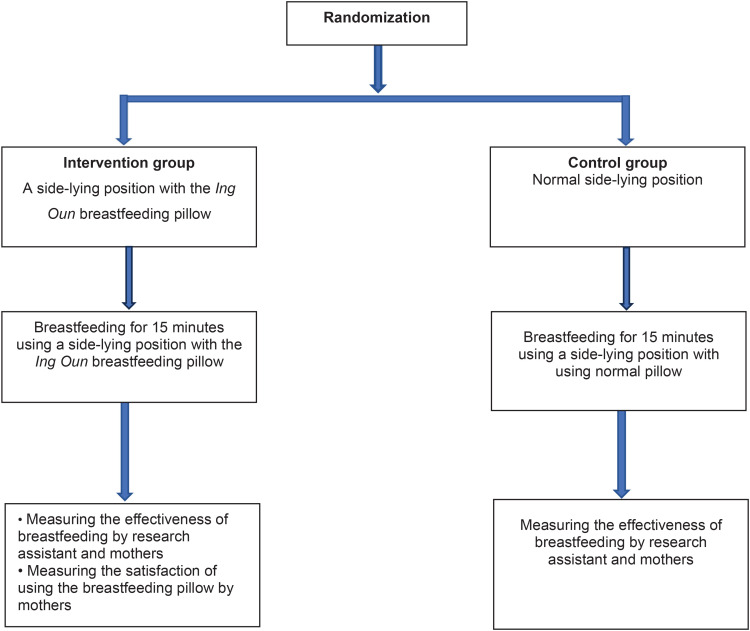
Recruitment flow chart in 2024, Postnatal Unit of Ramathibodi Hospital, Bangkok, Thailand

Among the 101 mothers who were initially assessed for eligibility in this study, 92 mothers met the inclusion criteria and consented to participate. All participants successfully completed the questionnaires, ensuring comprehensive data collection (Figure 3).

The participants were aged 18–33 years, with a mean age of 26.96 ± 2.56 years. The average postnatal day was 1.50 ± 0.55 days. The mean gestational age was 38.57 ± 0.96 weeks, and the mean baby birth weight was 2922.17 ± 304.92 g. All participants were first-time mothers with no previous breastfeeding experience. They received routine nursing care and newborn breastfeeding support provided by the hospital. No significant differences were found between the intervention and control groups in terms of descriptive characteristics (p>0.05), as shown in Table 1.

**Table 1:** Participant demographics (N=92)

*Characteristics*	*Intervention group* *(N=46)* *Mean (SD)*	*Control group* *(N=46)* *Mean (SD)*	*t*	*p*
**Age** (years)	26.96 (2.56)	26.26 (3.52)	1.09	0.28
**Postpartum** (days)	1.50 (0.55)	1.58 (0.65)	-0.69	0.53
**Gestational age** (weeks)	38.57 (0.96)	38.54 (0.86)	0.11	0.91
**Baby weight** (g)	2922.17 (304.92)	2881.52 (317.06)	0.63	0.49

### Effectiveness of breastfeeding by mothers

The data were not normally distributed (p<0.05); therefore, the Mann–Whitney U test was used for group comparisons. Mothers in the intervention group reported significantly less nipple pain during suckling (median=3, IQR: 2–4) compared with the control group (median=3, IQR: 1–4, U=674, p<0.001). The baby’s ability to continue suckling was also significantly higher in the intervention group (median=4, IQR: 2–4) than in the control group (median=3, IQR: 1–4, U=693, p<0.05). However, no significant differences were observed between the groups in securely holding the baby without slipping (median=3.5, IQR=2–4 vs median=3, IQR: 1–4, U=973, p=0.47) or holding the baby close to the mother’s body (median=4, IQR: 2–4 vs median=4, IQR: 1–4, U=939, p=0.27). Overall breastfeeding effectiveness scores were significantly higher in the intervention group (median=14, IQR: 8–16) compared with the control group (median=12, IQR: 7–16, U=675, p<0.05), as shown in Table 2.

**Table 2:** Comparison of breastfeeding effectiveness between the intervention and control groups by mothers in 2024, Postnatal Unit of Ramathibodi Hospital, Bangkok, Thailand (N=92)

*Effectiveness of breastfeeding*	*Intervention group* *(N=46)* *Median (IQR)*	*Control group* *(N=46)* *Median (IQR)*	*U*	*p*
Hold the baby tightly without slipping	3.5 (2–4)	3 (1–4)	973	0.47
The feeling of no pain at the nipples while the baby is suckling	3 (2–4)	3 (1–4)	674	<0.001
Baby can continue to suck	4 (2–4)	3 (1–4)	693	<0.05
Baby held close to mother’s body	4 (2–4)	4 (1–4)	939	0.27
Overall	14 (8–16)	12 (7–16)	675	<0.05

IQR: interquartile range.

**Table 3:** Comparison of breastfeeding effectiveness between the intervention and control groups by nurse-midwives in 2024, Postnatal Unit of Ramathibodi Hospital, Bangkok, Thailand (N=92)

*Effectiveness of breastfeeding*	*Intervention group* *(N=46)* *Median (IQR)*	*Control group* *(N=46)* *Median (IQR)*	*U*	p*
More areola seen above baby’s top lip	4 (3–4)	3 (1–4)	689	<0.001
Baby’s chin touches the breast	4 (2–4)	4 (2–4)	900	0.12
Baby’s head and body in line	4 (3–4)	3 (2–4)	526	<0.0001
Baby’s mouth open wide	4 (3–4)	4 (3–4)	897	0.09
Baby’s cheeks do not dimple during breastfeeding	4 (2–4)	4 (2–4)	999	0.57
Overall	20 (14–20)	18 (13–20)	551	<0.001

IQR: interquartile range.

### The effectiveness of breastfeeding by nurse-midwives

The data were analyzed and found to be not normally distributed (p<0.05), so the Mann-Whitney U test was used to assess significant differences. The results revealed a statistically significant difference in the effectiveness of breastfeeding by the nurse-midwives and a significant difference in the effects of signs of baby attachment during breastfeeding between normal side-lying breastfeeding and using the *Ing Oun* breastfeeding pillow in all outcomes. More specifically, the use of the *Ing Oun* breastfeeding pillow increased significantly, as greater areola was seen above the baby’s top lip (median=4, IQR: 3–4 vs median=3, IQR: 1–4, U=689, p<0.001) and the baby’s head and body in line (median=4, IQR: 3–4 vs median=3, IQR: 2–4, U=526, p<0.0001). However, no significant differences were found between the groups in terms of whether the baby’s chin touched the breast (median=4, IQR: 2–4 vs median=4, IQR: 2–4, U=900, p=0.12), the baby’s mouth opened wide (median=4, IQR: 3–4 vs median=4, IQR: 3–4, U=897, p=0.09) or the baby’s cheeks did not dimple during breastfeeding (median=4, IQR: 2–4 vs median=4, IQR: 2–4, U=999, p=0.57).

### Mothers’ satisfaction with the *Ing Oun* breastfeeding pillow

The results indicate a generally positive response from mothers regarding their satisfaction with the *Ing Oun* breastfeeding pillow. Many mothers found the pillow easy to use (50.0%) and appreciated its nonslip feature (63.0%). Half of the participants (50.0%) found the pillow easy to disassemble, whereas a majority (54.3%) noted that it was easy to clean. The design received a mixed response, with 41.3% of mothers finding it suitable and 54.3% somewhat satisfied. In terms of size and length, 47.8% of mothers were very satisfied, 50.0% were satisfied with their appearance, and 23.9% expressed some dissatisfaction. The fabric used was approved by 52.2% of the mothers, while 15.2% felt neutral about it.

With respect to the trialability of the pillow, 67.4% of the mothers reported that it effectively supported holding their baby during breastfeeding, and 56.5% reported that it was easy to carry their baby while using the pillow. In terms of physical comfort, 56.5% of mothers reported reduced arm pain, and 63.0% experienced less tension in their wrists while holding their baby. Finally, overall satisfaction with the innovation was high, with 65.2% of mothers pleased with its use for breastfeeding and an equal percentage (65.2%) feeling more confident in their breastfeeding ability when using the pillow. These results suggest that the innovative breastfeeding pillow was well received, particularly in terms of enhancing comfort and increasing confidence during breastfeeding, as presented in Table 4.

**Table 4:** Mothers’ satisfaction with the Ing Oun breastfeeding pillow in 2024, Postnatal Unit of Ramathibodi Hospital, Bangkok, Thailand (N=92)

*Satisfaction*	*Satisfaction levels (%)*				
	*Very satisfied*	*Satisfied*	*Neutral*	*Unsatisfied*	*Very unsatisfied*
**Easy-to-use features**					
Easy to use	50.0	50.0			
Non slippage	63.0	34.8	2.2		
Easy to disassemble	50.0	41.3	8.7		
Easy to clean	54.3	41.3	4.3		
**Suitability of equipment**					
Design	41.3	54.3	4.3		
Size and length	47.8	43.5	8.7		
Beauty	26.1	50.0	23.9		
Fabric used	30.4	52.2	15.2	2.2	
**Trialability**					
The ability to hold a baby to breastfeed	67.4	28.3	4.4		
Ease of carrying a baby	56.5	39.1	4.3		
Pain in the arm while holding the baby	56.5	41.3	2.2		
Tension in the wrist while holding the baby	63.0	32.6	4.3		
**Observability**					
Satisfaction with using innovation for breastfeeding	65.2	32.6	2.2		
Feelings of confidence in breastfeeding	65.2	32.6	2.2		

## DISCUSSION

This proof-of-concept study compared ergonomic support using the novel *Ing Oun* breastfeeding pillow in a side-lying position with the standard side-lying position among first-time mothers. The findings suggest that the *Ing Oun* breastfeeding pillow is feasible for supporting breastfeeding in first-time mothers. In line with the study hypothesis, mothers using the pillow showed improvements in indicators of breastfeeding effectiveness, including reduced nipple pain during suckling and an improved ability of the infant to continue suckling. Some indicators of attachment were also more favorable in the intervention group, such as greater visibility of the areola above the infant’s top lip and appropriate head–body alignment. In addition, mothers reported generally positive experiences when using the pillow. However, further evaluation and refinement of the pillow’s size, length, and overall appearance are needed.

Breastfeeding can be challenging for first-time mothers who have limited experience, and newborns may struggle with proper latching^19^. Discomfort during breastfeeding can make it difficult for mothers to adapt to their new role. In this study, mothers using the *Ing Oun* breastfeeding pillow reported reduced arm pain and wrist tension while holding their infants during breastfeeding. These observations suggest that the pillow may help improve maternal comfort during breastfeeding. This may be related to the ergonomic design of the pillow, particularly its weighted beaded interior, which may provide stability for supporting the infant’s body and help minimize movement during feeding. These findings align with those of previous studies indicating that breastfeeding pillows can reduce maternal discomfort by improving ergonomic positioning. For example, a randomized controlled trial by Sri Widiastuti et al.^7^ revealed that nursing pillows improved latch effectiveness and provided greater comfort for both mothers and babies. Similarly, Nisman et al.^20^ demonstrated that using a nursing pillow reduced maternal fatigue by maintaining a comfortable breastfeeding position. The findings of this study add to the existing evidence indicating that using a breastfeeding pillow, instead of relying solely on breastfeeding positions, can significantly help relieve maternal discomfort during breastfeeding for first-time mothers.

The side-lying breastfeeding position assists mothers in establishing breastfeeding, especially when fatigued^21^. Incorrect positioning and attachment are leading causes of nipple pain, with studies indicating that improper latching and infant positioning account for 89% of cases^22^. In this study, compared with the control group, the *Ing Oun* breastfeeding pillow group reported significant improvement in maternal comfort and reduced nipple pain. The ergonomic design of the pillow supports the infant’s head and promotes optimal alignment of the baby’s nose with the mother’s nipple, leading to a more effective latch. This improved alignment allows for a visible areola above the infant’s top lip, a key sign of proper latching^23^, contributing to reduced discomfort and enhanced breastfeeding experiences for the mothers. However, no significant differences were observed in some positioning aspects, such as the baby’s chin touching the breast and the width of the baby’s mouth during feeding. This may be related to the pillow being available in only one size, which may create a gap between the round pillow and the baby’s neck, making it less suitable for supporting the baby’s neck in some cases. Future refinement of the design is therefore needed to make the adjustable round pillow shapes more adaptable for infants of different sizes.

The pillow was also reported to improve the baby’s ability to continue sucking. This is important, as correcting positioning and attachment during the first few feeds has been associated with longer breastfeeding durations and fewer complications^22^. This study’s findings suggest that improved maternal comfort may also increase confidence in breastfeeding, a crucial factor in sustaining breastfeeding during the early postpartum period^24^. These promising initial findings serve as proof of concept for the *Ing Oun* breastfeeding pillow’s potential to improve maternal comfort and support breastfeeding practices. However, large-scale research is necessary to validate its efficacy and applicability.

The *Ing Oun* breastfeeding pillow was acceptable to participants and received high satisfaction, particularly for its ease-of-use features. Participants attributed these positive experiences to the pillow’s non-slip design and its ability to be easily disassembled and cleaned. Although the overall design of the pillow was considered suitable, they suggested that additional size and length options may improve its adaptability for infants of different sizes. Importantly, innovations should be designed to address user needs or solve specific problems, ultimately leading to better outcomes for users^21^. This suggests that while the pillow aids in positioning, its effectiveness may still depend on additional factors, such as the size and height of the baby, which were not considered in the current intervention. Additionally, the participants suggested offering more color options for the pillow. Since this is the first trial of the *Ing Oun* breastfeeding pillow, further studies are recommended to refine its design, such as developing variations in size and length and improving its user-friendliness to better accommodate a wider range of mothers and infants.

### Strengths and limitations

This study’s randomized controlled trial design is a key strength, as it minimizes the potential for confounding factors and reduces selection bias^25^. By including only first-time mothers, the study minimizes variability in breastfeeding practices. This limits bias related to prior experience, as these mothers are all experiencing breastfeeding and newborn care for the first time. However, a limitation of the study is the use of subjective data collection tools, such as the effectiveness of breastfeeding scales assessed by both nurses and mothers. These tools rely on observational data and were only applied after the intervention, without baseline measurements taken before the intervention. This limited the researcher’s ability to compare pre- and post-intervention outcomes. Another limitation is that breastfeeding effectiveness was assessed during a single breastfeeding session in the postpartum unit. As this study aimed to provide an initial proof-of-concept evaluation of the *Ing Oun* breastfeeding pillow, the intervention was applied only once to assess its immediate effect on breastfeeding positioning and attachment. Future studies should examine the effectiveness of the pillow across multiple breastfeeding sessions and over longer follow-up periods. Furthermore, while the study achieved a high response rate, likely due to mothers’ eagerness to use the *Ing Oun* breastfeeding pillow, the generalizability of the findings may be limited, as the study was conducted in a single hospital in Thailand. Further research in diverse settings is needed to confirm the broader applicability of these results.

## CONCLUSIONS

The *Ing Oun* breastfeeding pillow offers a potentially promising solution for improving breastfeeding. These findings contribute to the growing body of evidence that ergonomic aids can play a vital role in supporting breastfeeding mothers. However, further research is needed to refine the pillow design and explore its long-term impact on breastfeeding success.

## Supplementary Material



## Data Availability

The data supporting this research can be found in the Supplementary file.
